# Assessing mental health clinicians’ intentions to adopt evidence-based treatments: reliability and validity testing of the evidence-based treatment intentions scale

**DOI:** 10.1186/s13012-016-0417-3

**Published:** 2016-05-05

**Authors:** Nathaniel J. Williams

**Affiliations:** School of Social Work, Boise State University, 1910 University Dr., Boise, ID 83725-1940 USA

**Keywords:** Intention, Evidence-based treatment, Adoption, Mental health, Measurement

## Abstract

**Background:**

Intentions play a central role in numerous empirically supported theories of behavior and behavior change and have been identified as a potentially important antecedent to successful evidence-based treatment (EBT) implementation. Despite this, few measures of mental health clinicians’ EBT intentions exist and available measures have not been subject to thorough psychometric evaluation or testing. This paper evaluates the psychometric properties of the evidence-based treatment intentions (EBTI) scale, a new measure of mental health clinicians’ intentions to adopt EBTs.

**Methods:**

The study evaluates the reliability and validity of inferences made with the EBTI using multi-method, multi-informant criterion variables collected over 12 months from a sample of 197 mental health clinicians delivering services in 13 mental health agencies. Structural, predictive, and discriminant validity evidence is assessed.

**Results:**

Findings support the EBTI’s factor structure (*χ* ^*2*^ = 3.96, *df* = 5, *p* = .556) and internal consistency reliability (*α* = .80). Predictive validity evidence was provided by robust and significant associations between EBTI scores and clinicians’ observer-reported attendance at a voluntary EBT workshop at a 1-month follow-up (OR = 1.92, *p* < .05), self-reported EBT adoption at a 12-month follow-up (*R* ^2^ = .17, *p* < .001), and self-reported use of EBTs with clients at a 12-month follow-up (*R* ^2^ = .25, *p* < .001). Discriminant validity evidence was provided by small associations with clinicians’ concurrently measured psychological work climate perceptions of functionality (*R* ^2^ = .06, *p* < .05), engagement (*R* ^2^ = .06, *p* < .05), and stress (*R* ^2^ = .00, *ns*).

**Conclusions:**

The EBTI is a practical and theoretically grounded measure of mental health clinicians’ EBT intentions. Scores on the EBTI provide a basis for valid inferences regarding mental health clinicians’ intentions to adopt EBTs. Discussion focuses on research and practice applications.

**Electronic supplementary material:**

The online version of this article (doi:10.1186/s13012-016-0417-3) contains supplementary material, which is available to authorized users.

Research on evidence-based treatment (EBT) implementation in mental health settings is limited by a lack of reliable, valid, and practical measures to assess constructs likely to influence clinicians’ practice behaviors [1, 2]. One area in need of attention is the development of psychometrically sound measures of clinicians’ behavioral intentions with respect to EBT adoption. Intentions play a central role in numerous empirically supported theories of behavior and behavior change [3–5] such as the theory of reasoned action/theory of planned behavior [6, 7], social cognitive theory [8], Triandis’ attitude-behavior theory [9], and goal theory [10]. As a result, intentions have been identified, along with opportunities to act and the requisite ability, as a potentially important antecedent to successful EBT adoption [11, 12]. Despite this, few measures of mental health clinicians’ EBT intentions exist and the measures that have been used in individual studies have not been subject to thorough psychometric evaluation or testing [1, 12, 13]. This study addresses the need for a practical, theoretically grounded, and psychometrically sound measure of mental health clinicians’ intentions to adopt EBTs by evaluating the evidence-based treatment intentions (EBTI) scale.

## Background

Intentions represent self-instructions to engage in a targeted behavior or to obtain specific outcomes and as such capture the motivational factors that influence behavioral performance [9, 14]. Measures of intention reflect how hard a person is willing to try or how much effort they would exert to perform a behavior and therefore provide a numerical score that indexes the likelihood a person will perform or try to perform the behavior in question [3]. Several empirically supported theories of behavior and behavior change, including theories of attitude-behavior relations [6, 7], social cognitive theory [8], goal theory [10], and organizational behavior theories [15, 16], incorporate intentions as a primary determinant of behavior. These theories argue that intention represents the most proximal cause of behavior such that other influencing variables are either mediated by intention or serve as moderators of the intention-behavior relationship [17]. The validity of these predictions is supported by a meta-analysis of 422 studies which found an average correlation of *r* = .53 between intention and behavior [18] and by a meta-analysis of 47 experimental studies which found that an experimentally induced, medium-to-large increase in intention (*d* = .66) contributed to a small-to-medium change in salient behaviors (*d* = .36) [8]. Related meta-analyses focusing specifically on the relationship between healthcare professionals’ intentions and practice behaviors have demonstrated similarly strong effect sizes (*r* = .46) [19, 20]. Although many factors likely influence clinicians’ practice behaviors, these studies suggest that an understanding of mental health clinicians’ EBT adoption will be aided by the reliable and valid assessment of their EBT intentions [20].

Despite the robust theoretical and empirical support for the predictive efficacy of intentions, recent reviews of implementation research indicate that little attention has been paid to mental health clinicians’ EBT intentions. A systematic review of studies that examined healthcare professionals’ practice intentions and behaviors using the theory of planned behavior found only two of the 20 studies included mental health clinicians [12]. Two broader reviews of the relationship between healthcare professionals’ innovation-related intentions and practice behaviors reported similarly sparse attention to mental health practitioners and settings with one review reporting zero studies focused on mental health professionals [19] and the other indicating only four out of 76 studies included mental health professionals [20]. This is an important gap because there is evidence that the strongest individual-level predictors of behavior (e.g., intentions, self-efficacy) vary across professional disciplines, suggesting that the strength of the intention-behavior relationship may not generalize across healthcare provider populations and settings [12, 19, 20]. These reviews characterize research on mental health clinicians’ intentions to adopt EBTs as “extremely limited” and point toward the need for measurement instruments that support reliable and valid inferences in order to advance research on this theoretically and empirically important construct [12, 19, 20].

A measure of mental health clinicians’ intentions to adopt EBTs has important scientific implications for testing and extending individual-level theories of practice behavior change, such as research on clinicians’ attitudes toward EBTs [21, 22], as well as research on cross-level theories that link organizational social context and other inner and outer context variables to clinicians’ EBT adoption behavior [23, 24]. These theories posit that intentions form a mediational bridge that links attitudes to behavior and links higher-level contextual constructs such as organizational culture or climate to individual behavior. Testing these predictions requires a valid measure of EBT intentions [25].

A measure of clinicians’ EBT intentions also has practical value as an interim endpoint in intervention modeling studies and mechanism-focused randomized trials that seek to identify how implementation strategies work and can be optimized [19, 26]. In intervention modeling studies, key components of an intervention are manipulated in a small-scale simulation that mimics a real practice scenario, and outcomes are assessed using interim endpoints (such as intentions) rather than changes in professional behavior or clinical outcomes [19]. These studies reduce the time and expense required to identify the active components of implementation strategies; however, they depend on valid measures of intention that are highly correlated with the targeted behavior [19]. A valid measure of EBT intentions is therefore important for conducting such studies.

In mechanism-focused randomized controlled trials, investigators incorporate hypothesized mechanisms of change into the study’s measurement and design and use mediation analysis to test the extent to which hypothesized mechanisms accounted for the effects of the implementation strategy on the targeted outcome [26]. The ability to measure EBT intentions in this type of study allows investigators to assess the extent to which increased intention versus some other mechanism (e.g., decreased job-related barriers) accounted for the effects of the implementation strategy on the implementation outcomes [11, 23]. Such tests advance generalizable knowledge regarding the underlying mechanisms that are most salient for increasing EBT adoption in mental health settings and consequently accelerate the development of effective, efficient, and scalable implementation strategies [26].

Some investigators classify the intention to adopt an EBT as an implementation outcome in its own right, suggesting such a measure may be useful as a criterion variable in empirical and comparative effectiveness implementation studies [27]. For example, in their taxonomy of implementation outcomes, Proctor and colleagues [27] include the intention to adopt an EBT as an implementation outcome. They argue that this outcome occurs relatively early in the phased process of EBT exploration, adoption, implementation, and sustainment [28] and therefore likely has relevance for subsequent implementation outcomes such as EBT fidelity, penetration, and sustainment. Although their model differs from the theory of planned behavior [7] and from other behavioral science theories [3] in that they conflate intention with EBT adoption behavior, their taxonomy nonetheless highlights the range of outcomes that might be targeted by implementation strategies at different phases of the implementation process (e.g., exploration, preparation/adoption, implementation, sustainment) as well as the need for measures that support valid inferences regarding those outcomes in order to test the effectiveness of implementation strategies [28].

Three principles guided development of the EBTI. First, the EBTI was designed to assess mental health clinicians’ intentions to adopt EBT protocols supported by empirical research without unnecessarily limiting its focus to a single, historically bound intervention. Mental health services are characterized by a large number of EBTs applicable to a wide range of clinical presentations, the unknown need for (and anticipated future development of) new EBTs, and the necessity to incorporate client preference and clinical judgment into the EBT process [21, 27]. Given these considerations, a useful and generalizable measure of mental health clinicians’ EBT intentions must tap clinicians’ intentions to adopt empirically supported EBT protocols without focusing exclusively on a single, specific protocol. EBT intention was therefore defined as a clinician’s self-instruction to adopt or employ EBTs in her or his clinical practice with clients. The content domain refers to a clinician’s decision to adopt EBTs in practice and includes both declarative statements of self-instruction (e.g., “I intend to use an EBT in each session with clients”) as well as behavioral manifestations of self-instruction that represent preparatory steps necessary to enact the target behavior (e.g., obtaining manuals or training).

Second, in light of the multi-phase process of EBT implementation, the EBTI was designed to focus on clinicians’ intentions to *adopt* EBTs rather than their intentions to explore EBTs, achieve fidelity to EBTs, or sustain ongoing fidelity to EBTs [28]. Following Proctor and colleagues [27], adoption is defined in this study as a clinician’s action to try or employ an EBT with clients. Consistent with behavioral science theories [3, 17], adoption is conceptualized as a behavior (i.e., the act of employing an EBT with a client) that is distinct from, and is a consequent of, a clinician’s intention (i.e., self-instruction) to adopt an EBT. Emphasis on adoption as the behavioral referent for clinicians’ intentions (i.e., as opposed to other implementation outcomes that occur in different phases) was driven by the belief that it is the clinicians’ intentions to adopt an EBT that represents the pivotal decision point for changing clinicians’ practice behaviors [27, 28]. Forming an intention to adopt an EBT represents the end of the deliberative process involved in EBT exploration, and enacting that intention by actually adopting an EBT comprises an essential first step toward subsequent implementation outcomes of EBT fidelity and sustainment [28].

Third, the EBTI incorporates a novel approach to measuring clinicians’ EBT intentions by including behavioral indicator items (e.g., “I have recently attended trainings, workshops, supervision sessions, or other learning sessions focused on EBTs”) that signify clinicians’ concrete enactment of self-instructions to adopt an EBT in addition to generalized intention items (e.g., “I intend to use an EBT in each treatment session”) that characterize traditional measures of intention [3, 29]. The behavioral indicator items reflect clinicians’ self-instructions to adopt EBTs and are designed to augment the measurement of intention in a context where the behavior to be performed (i.e., EBT adoption) is relatively complex and may require significant preparatory steps before enactment (e.g., at a minimum, the clinician must obtain training in the EBT before adoption). Authoritative sources on the measurement of intention indicate that a valid measure must provide a continuous score that indicates the subjective likelihood a person will perform or try to perform the behavior in question [3]. From a theoretical standpoint, this score represents how hard the person is willing to try and how much effort he or she would exert to perform the behavior [7, 30]. Given the preparatory steps required to adopt an EBT and the potentially extended time lapse from a clinician’s initial formation of an intention to adopt an EBT to actual EBT adoption with clients, the behavioral indicator items are designed to increase the EBTI’s accuracy in indexing the likelihood a clinician will adopt an EBT.

Meta-analyses of the intention-behavior relationship indicate measures of intention that only capture a person’s general *desire* to perform a behavior are significantly less robust predictors of subsequent behavior than measures of intention that clearly assess the intensity and level of effort the person is prepared to exert to perform the behavior [30]. The behavioral indicator items are therefore designed to represent more stringent tests of clinicians’ intentions to adopt EBTs and consequently to provide separation between the scores of clinicians who possess a truly meaningful intention to adopt an EBT versus those who merely espouse a vague wish to adopt EBTs. Clinicians’ responses to the behavioral indicator items are hypothesized to arise from the same latent construct (i.e., intention) that drives their responses to the traditional intention items, and consequently, all the items are hypothesized to load on the same factor. Testing of the EBTI’s factor structure is important in order to assess the extent to which the five observed indicators tap a common underlying latent construct as hypothesized [31].

In addition to developing the EBTI and testing the reliability of EBTI scores, the present study also sought to develop structural, predictive, and discriminant-based evidence regarding the validity of inferences made with the EBTI. Two types of criterion variables were selected for this purpose. The first set of variables developed predictive validity evidence for inferences made with the EBTI. First, higher clinician EBT intentions at time 1 were expected to be moderately and positively associated with observed attendance at a voluntary EBT workshop 1 month after administration of the EBTI (time 2). Although many factors likely influence clinicians’ attendance at voluntary EBT workshops, the overall probability of attendance at a broadly applicable EBT workshop should be higher for the population of clinicians who intend to adopt EBTs in their practice compared to clinicians who lack such intentions. This variable therefore represented an objective criterion more likely to be manifest among clinicians with higher EBT intentions.

Second, consistent with the theory of planned behavior and other behavioral science theories [3, 6, 7, 17], higher EBT intentions were expected to exhibit a strong and positive relationship with clinicians’ EBT adoption behavior 12 months later (time 3). To the extent the EBTI assesses clinicians’ intentions to adopt EBTs, clinicians with higher scores should enact greater EBT adoption behavior. Third, higher EBTI scores were expected to exhibit the strongest positive association with clinicians’ self-reported use of EBTs with clients at time 3. Clinicians’ use of EBTs was conceptualized as an indicator of EBT adoption that captures clinicians’ action to try or employ an EBT in their work with clients. Accordingly, clinicians with higher intentions to adopt EBTs should have higher rates of EBT use with clients. This relationship represented the strongest concordance between the molarity of the EBTI and the criterion variable and was therefore expected to exhibit the strongest association.

The second set of variables provided discriminant-based validity evidence for inferences made with the EBTI. These variables assessed clinicians’ psychological climate perceptions of their work environment along three dimensions supported by prior research [32]. Psychological climate is an individual-level construct from the organizational research literature that describes clinicians’ perceptions of the impact of the work environment on their personal well-being [16, 32]. Psychological climate theory suggests clinicians’ perceptions of the support they receive in their work environment (i.e., functionality), their level of engagement with clients (i.e., engagement), and the extent to which they perceive their work environment as stressful (i.e., stress) represent constructs relevant to clinicians’ work experiences and practice behaviors but distinct from their intentions to adopt specific EBTs into practice [32, 33]. Consistent with this theory, prior research demonstrated small positive associations between clinicians’ attitudes toward EBTs and their psychological climate perceptions of functionality and engagement and generally negative and an overall non-significant association between clinicians’ EBT attitudes and their psychological climate perceptions of stress [34]. Given the theoretical and empirical link between attitudes and intentions [3, 17], the EBTI was expected to exhibit a similar pattern of relationships. Specifically, EBT intentions were expected to exhibit small positive associations with climate perceptions of functionality and engagement and a small negative association with stress. This pattern of relationships would provide discriminant-based validity evidence.

In sum, the present study assessed the reliability of scores on the EBTI and developed structural, predictive, and discriminant-based evidence of the validity of inferences made with the EBTI using multi-method, multi-informant criteria sequenced over a 12-month period. On the basis of the theory and research outlined above, scores on the EBTI were expected to exhibit (a) internal consistency and structural validity; (b) strong associations with clinicians’ prospectively measured attendance at a voluntary EBT workshop, number of EBTs adopted, and use of EBTs with clients; and (c) weak associations with concurrently collected measures of clinicians’ psychological climate perceptions of engagement, functionality, and stress.

## Methods

### Participants

The study included 197 mental health clinicians in 13 mental health agencies in a large midwestern city in the USA. Of the 216 clinicians who were present at the time the EBTI was administered, 197 completed the EBTI, representing a 91 % response rate (average number of clinicians per agency = 15, min = 6, max = 41). Clinicians delivered a range of clinical services (e.g., psychotherapy, family therapy, groups) in a variety of settings (e.g., school, clinic, home) using treatment models and procedures selected by clinicians. Table 1 provides a summary of participating clinicians’ demographic characteristics.

**Table 1:** Characteristics of participating clinicians (*n* = 197)

Characteristics	*n* (%)
Race	
White	162 (82)
African-American	18 (9)
“Other”	17 (9)
Ethnicity	
Hispanic	4 (2)
Non-Hispanic	193 (98)
Education	
No college	4 (2)
Associate degree	2 (1)
Bachelor’s degree	20 (10)
Master’s degree	166 (84)
Doctoral degree	5 (3)
Academic major of highest degree	
Education	26 (13)
Social work	79 (40)
Nursing	2 (1)
Psychology	28 (14)
Other/allied health	62 (32)
Gender	
Female	168 (85)
Male	29 (15)
Position	
Direct service provider	170 (86)
Supervisor	14 (7)
Neither	13 (7)
Age	
Mean (SD)	37.66 years (12.23)
Tenure with agency	
Mean (SD)	4.64 years (5.12)
Tenure in mental health settings	
Mean (SD)	10.16 years (9.06)

### Procedures

Clinicians were recruited for participation in the study during regularly scheduled staff meetings. Prior to the meetings, clinicians received either a memo or verbal information from agency administrators indicating that the agency’s executive director or CEO had approved the agency’s participation in the study and that clinicians were free to participate in the research if they wished. During the meetings, research assistants provided clinicians with verbal information and written informed consent documents that indicated that clinicians’ participation in the research was voluntary and that procedures were in place to ensure that agency administrators and supervisors would remain unaware of clinicians’ participation or non-participation. Following informed consent, clinicians completed surveys during staff meetings in which supervisors were not present. Research assistants unaffiliated with the agencies administered the surveys to increase clinicians’ confidence in the confidentiality of their responses. Survey collection occurred at two time points separated by 12 months. At the first time point (time 1), clinicians completed survey measures of their EBT intentions and psychological climate perceptions. Twelve months later (time 3), clinicians completed measures of their EBT adoption and EBT use with clients.

One month after time 1, all clinicians in participating agencies were invited to participate in an EBT workshop sponsored by the study (time 2). Clinicians were notified about the EBT workshop using agencies’ typical procedures for notifying employees of important information. Notifications included posted flyers on bulletin boards, announcements of the availability of the workshop at staff meetings, or via email. Clinicians’ attendance at the workshop was voluntary, and agency administrators were not informed of clinicians’ attendance or non-attendance. The EBT workshop was directed by nationally known experts in EBT who delivered training in an empirically supported modular cognitive behavior therapy protocol for youth [35]. This EBT addresses multiple mental disorders (i.e., anxiety, depression, trauma, and conduct problems) as well as their comorbid presentations and therefore had broad applicability to a range of clients seen by participating clinicians. The training was hosted by a well-established continuing education program at a local university. Clinicians’ attendance at the workshop was recorded by continuing education staff naïve to the study’s aims.

### Measures

#### Evidence-based treatment intentions

Clinicians’ intentions to adopt EBTs were measured using the EBTI. The EBTI was developed following steps outlined by DeVellis [31] including (a) definition of the latent construct to be measured (as described above), (b) development of an initial pool of 15 original items generated by the author using well-established procedures from the theory of planned behavior [29] and the principles described above, (c) vetting of the initial items by three implementation scientists with 60 years combined experience in mental health services, and (d) item refinement and selection based on iterative reviewer feedback. Reviewers evaluated each item’s clarity of expression as well as the extent to which it reflected the EBTI’s specified content domain. Items were reworded if any reviewer felt the meaning was unclear and items that failed to reflect the content domain were rejected based on the conservative criterion of a negative evaluation from any reviewer. This process resulted in a total of five items which were evaluated in the study and are presented in Table 2. Consistent with prior research on EBT adoption in children’s service systems [26], the instructions for the EBTI contextualized clinicians’ responses by defining an EBT as “a specific treatment protocol that has been developed through research and is supported by the results of controlled treatment studies.” Items were accompanied by a 7-point scale ranging from 1 (*strongly disagree*) to 7 (*strongly agree*) or an 11-point scale ranging from 0 to 10, as appropriate.

**Table 2:** Item-level means, standard deviations, ranges, and standardized CFA factor loadings

Evidence-based treatment intentions (EBTI) item	CFA factor loading	Mean	SD	Range
1. I have spoken with colleagues about their experiences with EBTs.	.67	4.69	1.63	1–7
2. I intend to use an EBT in each treatment session.	.88	4.75	1.58	1–7
3. I have recently attended trainings, workshops, supervision sessions, or other learning sessions focused on EBTs.	.48	5.08	1.75	1–7
4. I have searched the literature for appropriate EBTs to use with my clients.	.69	4.76	1.60	1–7
5. Out of the next 10 new clients you see, how many would you expect to treat using an EBT (0–10)?	.78	6.92	2.95	0–10

#### Evidence-based treatment workshop attendance

Clinicians’ attendance at the EBT workshop was observed and recorded by continuing education staff on-site during the workshop. Staff recorded the participation of each clinician who attended the workshop during a check-in period at the beginning of the training. Participating clinicians whose names and affiliations were on the check-in log for the workshop were coded as having attended the workshop. Clinicians who were working in participating agencies at the time of the workshop but whose names were not on the attendance log were identified as not attending the workshop. A total of 21 clinicians (12.8 %) who were employed in participating agencies at the time attended the EBT workshop.

#### Evidence-based treatment adoption

Clinicians’ EBT adoption was measured using nine items from the EBT checklist developed by Walrath et al. [36] and validated in previous research on EBT adoption in children’s mental health settings [37]. The nine EBTs were selected from the checklist a priori because of their applicability to the client populations served by the clinicians in this study and their reflection of the most up-to-date evidence. The selected EBTs included the following: cognitive behavioral therapy, behavioral parent training, contingency management, behavior therapy, relaxation training, self-control training, behavior modeling, and exposure/systematic desensitization. Clinicians indicated whether or not they had used each of the EBTs listed with any client in the last 6 months (*yes*, *no*). Clinicians’ responses to the nine items were summed to produce a continuous score indicating the number of EBTs adopted.

#### Evidence-based treatment use

Clinicians’ use of EBTs in their practice with clients was measured using a single broadband item, “What percentage of your clients do you currently treat using an EBT (0–100 %)?” To ensure continuity with the EBT intentions measure, the instructions provided an identical definition of evidence-based treatment emphasizing the use of specific protocols validated through research.

#### Psychological climate

Clinicians’ psychological climate perceptions were measured along three dimensions—engagement, functionality, and stress—using the organizational social context measure developed by Glisson and colleagues [32]. Structural and criterion-related evidence supporting the validity of inferences made with the OSC as well as score reliability has been provided by several studies in children’s service settings including two national samples and randomized trials [23, 32, 34, 38–41]. Climate perceptions of engagement indicate the extent to which clinicians’ perceive their work as personally meaningful and worthwhile and perceive themselves as able to remain personally involved with and concerned about clients. Climate perceptions of functionality indicate the extent to which clinicians’ perceive they receive the cooperation and support they need from coworkers and supervisors to do their jobs successfully and have a clear understanding of how they can be successful in the organization. Climate perceptions of stress indicate the extent to which clinicians’ perceive that they are emotionally exhausted from their work and unable to accomplish necessary tasks. Items on the OSC are accompanied by a scale ranging from 1 (*Never*) to 5 (*Always*). Alpha reliability for engagement (*α* = .79), functionality (*α* = .91), and stress (*α* = .94) were above recommended cutoffs in the present study.

### Data analysis

Structural validity evidence for inferences based on the EBTI was developed using confirmatory factor analysis (CFA). Models were implemented with Mplus [42] statistical software (version 7). The CFA models accounted for the nested structure of the data (i.e., clinicians within agencies) using maximum likelihood estimation with robust standard errors (MLR) and the Satorra-Bentler scaled chi-square correction for the model chi-square test. Missing data were imputed using full information maximum likelihood (FIML) estimation [42]. Model fit was assessed using the chi-square model test statistic, and three goodness-of-fit indices: the comparative fit index (CFI), the root-mean-square error of approximation (RMSEA), and the standardized root-mean-square residual (SRMR) [43]. Good model fit is supported by a failure to reject the null hypothesis of the chi-square model test (i.e., *p* ≥ .05) and by values of CFI ≥.95, RMSEA ≤.05, and SRMR ≤.08 [43].

Three CFA models were tested. The first examined a single factor model in which all five EBTI items loaded on a single latent construct. Good model fit in this analysis indicates that the EBTI items measure a common latent construct. The second CFA included items assessing both EBT intentions (i.e., the EBTI) and EBT adoption (i.e., the EBT checklist). This analysis tested a two-factor model in which each set of items loaded on distinct latent constructs (i.e., intentions vs. adoption). Because of the dichotomous indicators included in the EBT checklist, this analysis used a robust weighted least squares estimator (WLSMV). Good model fit from this analysis provides additional support for the factor structure of the EBTI as well as discriminant validity evidence with respect to EBT adoption. The third CFA tested a single factor model in which all EBTI and EBT checklist items were forced to load on a single factor. This analysis also relied on the WLSMV estimator. Poor model fit from this analysis provides additional structural validity evidence by indicating that the two sets of items are not derived from a common underlying latent construct [43]. A model chi-square difference test was conducted comparing the one- and two-factor models to provide further structural and discriminant validity evidence. A statistically significant model chi-square difference test supports the two-factor model and indicates EBT intentions and EBT adoption items load on separate latent constructs.

The internal consistency reliability of scores on the EBTI was tested using Cronbach’s alpha. Predictive and discriminant validity evidence for inferences made with the EBTI was developed using two-level mixed effects regression models (also known as hierarchical linear models) implemented via HLM software, version 6.08 [44]. These models incorporated random agency intercepts to account for the nesting of clinicians within agencies [45]. A logit link function was incorporated for models examining the dichotomous outcome workshop attendance (i.e., two-level mixed effects logistic regression model). In models developing predictive validity evidence, the criterion variables were modeled as a function of clinicians’ EBT intentions and potential confounding variables including clinician education, position (supervisor vs. front-line clinician), and years of experience. In models developing discriminant-based validity evidence, clinicians’ psychological climate perceptions were modeled as a function of EBT intentions and clinician covariates. The variance accounted for by each model was calculated as (*σ* ^2^_Null_ − *σ* ^2^_H1_)/*σ* ^2^_Null_, where *σ* ^2^_Null_ represents the clinician-level criterion variance in the unconditional model (i.e., the model with no predictors) and *σ* ^2^_H1_ represents the unexplained clinician-level criterion variance in the conditional model (i.e., the model with the EBTI) [45]. For the two-level mixed effects logistic regression model, an odds ratio was calculated as a measure of effect size [45].

## Results

### Structural validity evidence

The first CFA tested the hypothesized one-factor model in which all five EBT intention items loaded on a single latent construct. This model demonstrated excellent fit as indicated by the non-significant model chi-square test (*χ* ^*2*^ = 3.96, *df* = 5, *p* = .556) and favorable values of the approximate fit indices (RMSEA = .00, *p* RSMEA < .05 = .77, CFI = 1.00, SRMR = .02). All items loaded onto one factor and all item loadings were statistically significant (*p*’s < .001) with an average standardized loading of .70 (see Table 2). Based on these results, the one-factor model was accepted without modification.

The second CFA incorporated items from both the EBTI and the EBT checklist and tested the hypothesized two-factor model. In this analysis, the EBTI items were constrained to load on a single latent factor and the EBT checklist items were constrained to load on a second latent factor. The model demonstrated good fit to the data as indicated by the model chi-square test (*χ* ^*2*^ = 99.77, *df* = 76, *p* = .035), favorable values on the approximate fit indices (RMSEA = .04, *p* RSMEA < .05 = .78, CFI = .94), and high standardized factor loadings on both scales (EBTI mean loading = .70; EBT checklist mean loading = .74). The latent variable correlation between the EBTI and the EBT checklist in this model was *r* = .47.

The third CFA incorporated all of the EBTI and EBT checklist items and tested a one-factor model in which all items loaded on a single latent construct. This model demonstrated significantly worse fit to the data as indicated by a statistically significant model chi-square difference test (*χ* ^*2*^ = 44.24, *df* = 1, *p* = .000) and substantially worsened values on the model chi-square test (*χ* ^*2*^ = 156.59, *df* = 77, *p* = .000) and approximate fit indices (RMSEA = .07, *p* RSMEA < .05 = .01, CFI = .80). Together, these results provide structural evidence to support the validity of inferences made with the EBTI. Results indicate the five EBTI items measure a common underlying latent construct that is distinct from the latent construct of EBT adoption as measured by the EBT checklist.

### Reliability

Cronbach’s alpha for the EBTI was *α* = .80 indicating good internal consistency among scores on the five items. Examination of the item-level means and distributions revealed good item-level variances with means near the center of the item ranges and approximately normal distributions (see Table 2).

### Predictive validity evidence

Table 3 presents results from the two-level mixed effects regression analyses linking the EBTI to each of the EBT criterion variables across the two waves of data collection. The pattern of relationships provides predictive evidence that supports the validity of inferences made with the EBTI. As expected, clinicians who reported higher EBT intentions at time 1 had significantly higher odds of attending an EBT workshop at time 2 (*γ* = .65, SE = .30, *p* = .033, OR = 1.92). For every one point increase in clinicians’ EBT intentions as measured by the EBTI, clinicians’ odds of attending the EBT workshop increased by 92 %. An even stronger relationship was observed between increased EBT intentions and increased EBT adoption (*γ* = .55, SE = .15, *p* = .001), with EBTI scores accounting for 17 % of the variance in EBT adoption. The strongest relationship was observed between clinicians’ EBT intentions and EBT use (*γ* = 11.80, SE = 1.83, *p* < .001) with EBTI scores accounting for 25 % of the variance in EBT use. These relationships were stronger than those observed for the psychological climate criterion variables (described below) despite being collected 1 or 12 months later. These results provide predictive evidence to support the validity of inferences made with the EBTI.

**Table 3:** Relations between clinicians’ EBTI scores and EBT workshop attendance, EBT adoption, and EBT use

	EBT workshop attendance (1 month)		EBT adoption (12 months)		EBT use (12 months)	
Variable	Coeff.	SE	Coeff.	SE	Coeff.	SE
Intercept	−2.75**	.76	4.98***	.36	66.06***	4.89
Years of experience	−.02	.04	.08**	.03	.15	.33
Education	1.20	1.01	.49	.77	−.23	9.40
Position	−1.16	1.30	.59	.38	−.90	4.55
EBTI (EBT intentions)	.65*	.30	.55***	.15	11.80***	1.83
Agency intercept variance	4.10		1.04		212.89	
Clinician-level variance	–		4.21		561.58	
Pseudo-*R* ^2^	–		.17		.25	

*Note*: These are two-level mixed effects regression analyses with random agency intercepts. The model for workshop attendance is a two-level mixed effects logistic regression model to account for the binary outcome. Due to attrition and missing values, *n*s range from *n* = 164 (workshop attendance) to *n* = 100 (EBT use)

***p ≤ .001; **p < .01; *p < .05

Table 4 provides a closer look at the relationship between EBTI scores and clinicians’ EBT workshop attendance by comparing item means for clinicians who attended and did not attend the EBT workshop. Clinicians who attended the EBT workshop scored significantly higher on all EBTI items with standardized mean differences ranging from *d* = .50 to *d* = .74. The strongest mean difference between clinicians who attended the EBT workshop and those who did not was on the EBTI item referring to clinicians’ recent participation in an EBT-focused learning session such as a workshop. These results provide additional validity evidence to support inferences made with the EBTI.

**Table 4:** Item means for EBT workshop attenders and non-attenders

	Attended EBT workshop		Did not attend EBT workshop		Standardized mean difference
EBTI item	M	SD	M	SD	(d)
Spoken with colleagues about their experiences	5.75	1.29	4.68	1.51	.70**
Intend to use in each session	5.45	1.36	4.69	1.51	.50*
Recently attended learning sessions	6.25	1.16	4.99	1.71	.74**
Searched the literature	5.45	1.23	4.66	1.56	.51*
Number of new clients expect to treat with EBT (0–10)	8.30	2.36	6.64	2.97	.56*

**p < .01; *p < .05

### Discriminant validity evidence

Table 5 presents the two-level mixed effects regression analyses linking EBTI scores with clinicians’ concurrently measured psychological climate perceptions. Consistent with expectations, the EBTI exhibited weak relationships with clinicians’ climate perceptions of functionality (*γ* = 1.26, SE = .40, *p* = .003) and engagement (*γ* = .43, SE = .20, *p* = .037) and a non-significant relationship with stress (*γ* = −.86, SE = .61, *p* = .161). The percentage of variance explained by the EBTI was small for functionality (6 %), engagement (6 %), and stress (<1 %). These relationships were weaker than the EBTI’s associations with the EBT criteria described above despite being collected at the same time as the EBTI and via the same method. Overall, this pattern of relationships provides discriminant validity evidence to support inferences made with the EBTI, in line with expectations and prior research.

**Table 5:** Relations between clinicians’ EBTI scores and psychological work climate perceptions

	Functionality		Engagement		Stress	
Variable	Coeff.	SE	Coeff.	SE	Coeff.	SE
Intercept	51.09***	1.10	45.02***	.36	56.30***	1.69
Years of experience	.14	.07	.15***	.04	−.28*	.11
Education	−2.06	1.11	−.83	.55	2.09	1.67
Position	3.24	2.17	−.58	1.12	−7.07*	3.27
EBT intentions (EBTI)	1.26**	.40	.43*	.20	−.86	.61
Agency intercept variance	10.11		.42		24.46	
Clinician-level variance	61.82		16.79		139.35	
Pseudo-*R* ^2^	.06		.06		.00	

*Note*: *n* = 195. These are two-level mixed effects regression analyses with random agency intercepts

***p < .001; **p < .01; *p < .05

## Discussion

The goal of this study was to assess a new measure of mental health clinicians’ intentions to adopt EBTs. Building on empirically supported behavioral science theories [3], items for the EBTI were designed to assess the strength of clinicians’ intentions to adopt EBTs in their work with clients. Results confirm the selected items tap a common underlying latent construct. Consistent with the theory of planned behavior and other behavioral science theories [3, 6–10], clinicians who reported higher intentions to adopt EBTs at baseline were more likely to voluntarily attend an EBT workshop 1 month later and to report greater adoption and use of EBTs in their work with clients 12 months later. The higher number of EBTs adopted and the greater use of EBTs with clients suggest the EBTI is capturing clinicians’ intentions to adopt EBTs in their practice. The small associations between the EBTI and clinicians’ concurrently measured psychological climate perceptions of engagement, functionality, and stress provide discriminant validity evidence to support inferences made with the EBTI. These relationships suggest that EBTI scores capture something meaningful about clinicians’ intended EBT adoption rather than assessing their more generalized perceptions of engagement with clients, support from colleagues in the workplace, or levels of stress in their professional roles. Together, these results support the validity of inferences made with the EBTI regarding mental health clinicians’ intentions to adopt EBTs. The EBTI and scoring instructions can be found in Additional file 1.

A measure that supports valid inferences regarding mental health clinicians’ EBT intentions is important because of the central role that individual clinicians play in the EBT implementation process and the importance of intention to clinicians’ volitional behaviors [14, 19, 20]. The EBTI provides a broadly applicable tool that allows investigators to assess clinicians’ intentions to adopt EBTs and therefore may be used to advance research on the individual-level determinants of EBT adoption, the cross-level mechanisms through which contextual factors influence EBT adoption, and as a criterion variable in intervention modeling studies or mechanism-focused randomized trials [19, 26, 27].

The EBTI also has practical value for aiding stakeholders and researchers in assessing the intentions of targeted populations of mental health clinicians to adopt EBTs. Suboptimal rates of EBT adoption may be observed in service systems for a variety of reasons; however, well-established theoretical models from the behavioral sciences (e.g., the theory of reasoned action/theory of planned behavior, social cognitive theory, Triandis’ theory of attitude-behavior relations [3]) as well as more recently formulated implementation science theories (e.g., the capability, opportunity, and motivation or COM-B model [11]) argue that intention represents an important component of one of three primary determinants of clinicians’ practice behaviors: capability, opportunity, and motivation [11]. Although some theories construe intention as only one facet of the broader construct of motivation, the EBTI nonetheless provides investigators and other stakeholders with a reliable and valid tool to assess this important facet [3, 11].

Limitations of the present study and directions for future research focus on several areas. First, because of the complexity of mental health treatment and the importance of incorporating client preference and clinician judgment into the EBT process, the EBTI focuses broadly on the adoption of EBTs for a wide range of clinical presentations. The strong-to-moderate association of the EBTI with an inventory of specific EBTs suggests clinicians associated the EBTI with their intentions to adopt specific EBT protocols. While these results provide criterion-related validity evidence, this broad focus is not informative for specific targeted EBTs. In cases where systems or research are focused on implementing specific EBTs, the EBTI might be reworded to focus on specific EBT protocols (e.g., parent-child interaction therapy). Although such adaptations might be theoretically meaningful and empirically supported, additional psychometric work would be necessary to support this hypothesis. For example, the EBTI items could be modified to assess clinicians’ intentions to adopt a specific EBT following workshop training. Theory and empirical research suggest increased specificity of the behavioral target (i.e., a specific EBT) should strengthen the relationship between scores on the EBTI and prospectively measured criterion variables. However, changes to item wording or referent may influence the EBTI’s factor structure and the validity of inferences made with the EBTI in unanticipated ways, thereby requiring additional evaluation.

Second, the EBTI’s innovative approach to measuring EBT intentions may have implications for its validity in predicting clinicians’ adoption of specific EBTs. Given the complexity of adopting an EBT and the preparatory steps required to perform this behavior, the EBTI incorporates behavioral indicator items that extend the traditional approach to measuring intentions [29]. Although results of this study supported this approach by demonstrating that the behavioral indicator items tap the same latent construct as the traditional intention items, behavioral indicator items may influence the utility and predictive validity of inferences made with the EBTI once translated to other contexts. For example, many mental health treatments are complex and require significant training, whereas other treatments, such as measurement-based care, may be simpler by comparison. It is unclear whether scores on the EBTI would similarly predict both types of behaviors or if perhaps modifications to the EBTI items may enhance its utility for predicting specific EBTs. The relatively lower factor loading of the item “I have recently attended trainings, workshops, supervision sessions, or other learning sessions focused on EBTs” suggests that the complexity of the EBT to be adopted may have implications for the utility of some of the EBTI items.

The tension between general and specific EBT intention items highlighted here parallels a broader dynamic within the field of implementation science regarding the generality versus specificity of implementation outcomes and the factors that influence those outcomes. A molecular approach to implementation research assumes implementation outcomes such as EBT adoption, fidelity, and sustainment and the factors that influence those outcomes (such as EBT intentions) are EBT-specific. This approach requires the development and validity testing of measures of constructs such as EBT intentions, EBT attitudes, or implementation climate for each of the specific EBTs relevant to the variety of clients seen in mental health service settings. Examples of this molecular conceptualization include work by organizational theorists such as Klein and Sorra [46] and Weiner and colleagues [47] who have developed the construct of implementation climate (focusing exclusively on the climate for a single innovation) and traditional approaches to the constructs of intention and attitudes [29]. Although this approach may be attractive from a research design perspective (i.e., holding the EBT constant in the design eliminates one potential confounding factor), its generalizability to typical mental health service settings and its utility for the study of EBT implementation in usual care may be limited because of the variety of patients typically seen in practice and the range of EBTs applicable to their needs. This approach seems most applicable to EBTs that have the broadest application to the full spectrum of client populations seen in mental health practices (e.g., measurement-based care [48]).

A contrasting and more generalized approach to implementation research suggests implementation outcomes and the factors that influence those outcomes may be generalizable across classes of EBTs (e.g., psychotherapy models) that share salient features (e.g., the use of specific interpersonal behaviors in an interactional process with a client) and can be used interchangeably within the same practice encounter (e.g., a mental health clinician may select interpersonal psychotherapy for depression or cognitive behavioral therapy for depression but will likely use either EBT in the same weekly 50-min session format). This approach invokes a molar as opposed to molecular level of specificity. The molar approach is consistent with the definition of EBT articulated by the American Psychological Association [49] and other professional associations which not only focuses on the use of empirically validated interventions (i.e., EBTs) in practice settings but also stresses the importance of patient choice in treatment selection and the use of clinician expertise.

Representative constructs from this molar approach include EBT implementation climate as developed by Ehrhart and colleagues [33], EBT attitudes as conceptualized in the widely cited EBPAS measure [21], and the EBTI. These molar measures of implementation-related constructs seek to address the range of EBTs applicable to mental health settings. The molar approach has practical advantages in terms of reflecting real world practice, permitting investigators to study a variety of EBTs and generalizing results to routine practice settings. From a validity testing perspective, however, important questions remain regarding the overlap between molar and molecular constructs as well as the incremental predictive value of these measures for various implementation outcomes. Direct, head-to-head comparisons of these measures will likely provide fruitful information for advancing our understanding of the generality and specificity of factors that influence EBT outcomes.

Third, the EBTI was applied in the present study to clinicians delivering youth mental health services. Studies that develop validity evidence for inferences based on the EBTI in adult mental health services are needed. In addition, other service sectors such as public health, child welfare, substance abuse, and social services are moving to increase the use of EBTs in service delivery and the EBTI may be useful for these settings in both research and practice contexts [27, 28].

Fourth, the present study was conducted within the context of large-scale behavioral system change, and it is possible that this context influenced the results. For example, clinicians may have been sensitized to the importance or challenges of EBT adoption and this may have increased or decreased their intentions to adopt EBTs. Future research that examines clinicians’ EBT intentions in larger samples will be informative for developing normative data on clinicians’ EBT intentions and for understanding how clinicians’ EBT intentions vary across regions and providers.

Fifth, additional studies are needed to further evaluate the psychometric characteristics of the EBTI using alternate-method criterion variables. Although the results of this study supported the conceptual distinction between EBT intentions as measured by the EBTI and EBT adoption as measured by the EBT checklist, the single item measure of EBT use did not permit examination of the uniqueness of the intention and use constructs. It is possible that the EBTI captures both intention and use latent constructs. While a strong association between intention and subsequent behavior is typically viewed as a strength of the intentions construct for research purposes, this strength presents a barrier to providing discriminant-based evidence of validity for measures. An additional limitation is the use of clinician self-report measures for all constructs in the study with the exception of workshop attendance. The use of multiple self-report measures introduces the possibility of inflated correlations due to common method bias [50]. The 12-month time span between measurement occasions provides some protection against this by reducing the salience of clinicians’ earlier responses and by impeding the retrieval of prior responses from memory [50]. Finally, the definition of EBT adoption used in this study did not differentiate between new and ongoing EBT adoption, an issue that appears unresolved in the larger literature [27]. If clinicians were already using EBTs at time 1, the post hoc assessment of intentions would likely be biased by these behaviors. Results of this study provide evidence supporting the EBTI’s association with EBT adoption and use; however, future studies should examine the association between EBTI scores and clinicians’ new adoption of EBTs.

## Conclusions

Mental health clinicians’ intention to adopt EBTs represents a potentially important antecedent for increasing EBT implementation in mental health systems. The present study provides a first step toward making reliable and valid inferences regarding clinicians’ EBT intentions using a brief and practical measure. Results provide structural, predictive, and discriminant-based evidence to support the validity of inferences made with the EBTI. Findings from this study highlight several directions for future research and provide a practical tool for stakeholders seeking to understand the factors at multiple levels that influence EBT adoption in mental health services.

## Additional file

Additional file 1: Evidence-based treatment intentions scale (EBTI). (DOCX 16 kb)
