# Supplementary material for: Assessing mental health clinicians’ intentions to adopt evidence-based treatments: reliability and validity testing of the evidence-based treatment intentions scale
Source: Implement Sci. 2016 May 5;11:60. doi: 10.1186/s13012-016-0417-3 (PMC4857292; doi:10.1186/s13012-016-0417-3)
Supplement: Additional file 1: — Evidence-based treatment intentions scale (EBTI). (DOCX 16 kb) [file 13012_2016_417_MOESM1_ESM.docx]

**Evidence-Based Treatment Intentions Scale (EBTI)**

Nathaniel J. Williams

natewilliams@boisestate.edu

This 5-item scale assesses mental health clinicians’ intentions to adopt evidence-based treatments in their practice with clients. Intention is defined as a commitment or self-instruction to engage in a targeted behavior and captures the motivational factors that influence evidence-based treatment adoption.

Instructions: Please indicate the extent to which you agree with each statement. The term “evidence-based treatment” or EBT refers to “*a specific treatment protocol that has been developed through research and is supported by the results of controlled treatment studies*.”

SD = Strongly Disagree N = Neither agree nor disagree SA = Strongly agree

|  | SD N SA |
| --- | --- |
| 1. I have spoken with colleagues about their experiences with EBTs. | 1 2 3 4 5 6 7 |
| 1. I have recently attended trainings, workshops, supervision sessions, or other learning sessions focused on EBTs. | 1 2 3 4 5 6 7 |
| 1. I intend to use an EBT in each treatment session. | 1 2 3 4 5 6 7 |
| 1. I have searched the literature for appropriate EBTs to use with my clients. | 1 2 3 4 5 6 7 |
| 1. Out of the next 10 new clients you see, how many would you expect to treat using an EBT? | 0 1 2 3 4 7 8 9 10 |

**SCORING INSTRUCTIONS**

The scale score is computed by taking the mean of the five items. Scales with less than 4 completed items (i.e., 80% of items) should not be scored.

Please direct any questions to Dr. Williams via email at natewilliams@boisestate.edu.
